# Modelling the national economic burden of non-surgical periodontal management in specialist clinics in Malaysia using a markov model

**DOI:** 10.1186/s12903-024-04094-z

**Published:** 2024-03-18

**Authors:** Ainol Haniza Kherul Anuwar, Chiu Wan Ng, Syarida Hasnur Safii, Roslan Saub, Norintan Ab-Murat

**Affiliations:** 1 Department of Community Oral Health and Clinical Prevention, Faculty of Dentistry, Universiti Malaya, Kuala Lumpur, 50603 Malaysia; 2 Department of Social and Preventive Medicine, Faculty of Medicine, Universiti Malaya, Kuala Lumpur, 50603 Malaysia; 3 Department of Restorative Dentistry, Faculty of Dentistry, Universiti Malaya, Kuala Lumpur, 50603 Malaysia

**Keywords:** Periodontitis, Non-surgical periodontal treatment, Health economic, Economic burden, Cost

## Abstract

**Background:**

Non-surgical periodontal treatment is the mainstay of periodontal treatment. In Malaysia, the prevalence of periodontal disease is substantial among adults with almost half of them having periodontitis. Therefore, we estimated the economic burden of non-surgical periodontal treatment in specialist clinics in Malaysia.

**Methods:**

Relevant data from multiple data sources which include national oral health and health surveys, national census, extensive systematic literature reviews, as well as discussion with experts, were used to estimate the economic burden of non-surgical periodontal management in specialist clinics in Malaysia in 2020. This estimation was done from the oral healthcare provider’s perspective in both public and private sectors using an irreducible Markov model of 3-month cycle length over a time horizon of one year.

**Results:**

In 2020, the national economic burden of non-surgical periodontal treatment during the first year of periodontal management in specialist clinics in Malaysia was MYR 696 million (USD 166 million), ranging from MYR 471 million (USD 112 million) to MYR 922 million (USD 220 million). Of these, a total of MYR 485 million (USD 115 million) and MYR 211 million (USD 50 million) were the direct oral healthcare cost in public and private dental clinics, respectively.

**Conclusion:**

The findings of this study demonstrated substantial economic burden of non-surgical periodontal management in specialist clinics in Malaysia. Being a life-long disease, these findings highlight the importance of enforcing primary and secondary preventive measures. On the strength and reliability of this economic evidence, this study provides vital information to inform policy- and decision-making regarding the future direction of managing periodontitis in Malaysia.

**Supplementary Information:**

The online version contains supplementary material available at 10.1186/s12903-024-04094-z.

## Introduction

In Malaysia, the prevalence of periodontal disease is substantial (94%) among adults aged 15 years and above with 48.5% of them having periodontitis. Concerning the severe form of periodontitis, the prevalence was higher at 18.2% as compared to the global prevalence [1]. It affects mainly those aged 45–54 years old (29.4%) and the prevalence subsequently decreases with age. The prevalence of severe periodontitis among Malaysian adolescents (15–19 years old) has increased by 30-fold in a decade from the year 2000 to 2010 [1]. This trend is worrying and poses a great challenge to the healthcare provider as adolescents are most likely to be exposed to and adopt risky behaviours (e.g., smoking, and high sugar diet), which may lead to further increase in the prevalence and progression of the disease [2]. Patients with periodontitis, especially those with severe forms, may require complex periodontal treatment. With the availability of various treatment modalities, treating periodontitis may have substantial clinical and financial consequences. Furthermore, this disease will cause oral disability, contributes to social inequality, and negatively affect quality of life in some cases [3, 4] This issue may result from marginalisation of oral healthcare from the mainstream healthcare [5], as well as lower priority being given to the management of periodontitis as compared with other oral diseases such as dental caries [6].

Malaysia adopts dual healthcare system provided by public and private sectors. The public sectors are highly subsidised from taxation with minimum user fee whilst private sectors depend on the out-of-pocket payment (OOP) [7]. Like many other countries, Malaysia has limited healthcare resources. Therefore, economic evidence is essential to facilitate evidence-based decision making by providing data to identify, measure, and compare resource allocation with impact, scalability, and sustainability of interventions in optimising population health [8]. Based on this evidence, an effective and economically defendable public health interventions can be established to produce a sustainable change. This is especially important in managing periodontal disease in Malaysia. Although this disease can be prevented, diagnosed easily, and treated effectively, the prevalence of this disease continues to rise, and to make matters worse, the impact of periodontitis may go beyond oral cavity. Through haematogenous dissemination of both bacteria and bacterial products, periodontitis interacts with various systemic diseases, such as diabetes, atherosclerosis, rheumatoid arthritis, and pulmonary infections [9]. Conversely, having non-communicable disease that shares the same modifiable risk factors will make individuals with such diseases susceptible to periodontitis. This bidirectional relationship between periodontitis and non-communicable diseases (NCDs) particularly diabetes [10], will inflict substantial socioeconomic impact on the country.

Comprehensive periodontal therapy involves management of patients throughout their lifetime, which mainly involved non-surgical periodontal treatment (NSPT). These patients may at any point of time develop recurrence of disease due to various factors (e.g., smoking, and sub-optimally controlled diabetes) associated with their susceptibility to periodontitis [11]. If this happens, patients may need to be re-treated, and this will have significant financial implications on both providers and patients [12]. Thus, it is important to highlight the impact of providing such treatment in monetary value so it can be valued by the policy and decision makers. It is also essential to economically justify the importance of having efficient policy and effective interventions to tackle this disease and its related diseases. Additionally, accurate knowledge of managing this disease can assist the stakeholders in formulating and prioritising healthcare policies or interventions, as well as in distributing limited resources to achieve policy efficiency. Therefore, in this study, economic arguments using multiple data sources were developed to inform policymaking for management of periodontitis in Malaysian dental facilities.

## Materials and methods

### Overview

To estimate the economic burden of non-surgical periodontal management in specialist clinics in Malaysia in year 2020, the required information was the clinical burden of the disease, patterns of oral healthcare services utilisation, treatment outcomes (i.e., transitional probabilities), and direct medical care costs. Using this information, the economic burden was estimated from the perspective of public and private oral healthcare providers using an irreducible Markov model.

### Estimating clinical burden of periodontitis among adults with periodontitis in Malaysia

Multiple data sources were used to estimate the clinical burden of periodontitis among adults aged 15 years and above in Malaysia. The prevalence of periodontitis was obtained from the 2010 National Oral Health Survey for Adults (NOHSA) [1]. National Oral Health Survey is conducted once every 10 years by the Oral Health Programme, Ministry of Health Malaysia aimed to determine the oral health status of adult population and future oral healthcare needs of the population. However, due to COVID-19 pandemic, the decennial NOHSA for 2020 was postponed and conducted in late 2022. To date, the findings of the national survey have yet to be published.

**Fig. 1:**
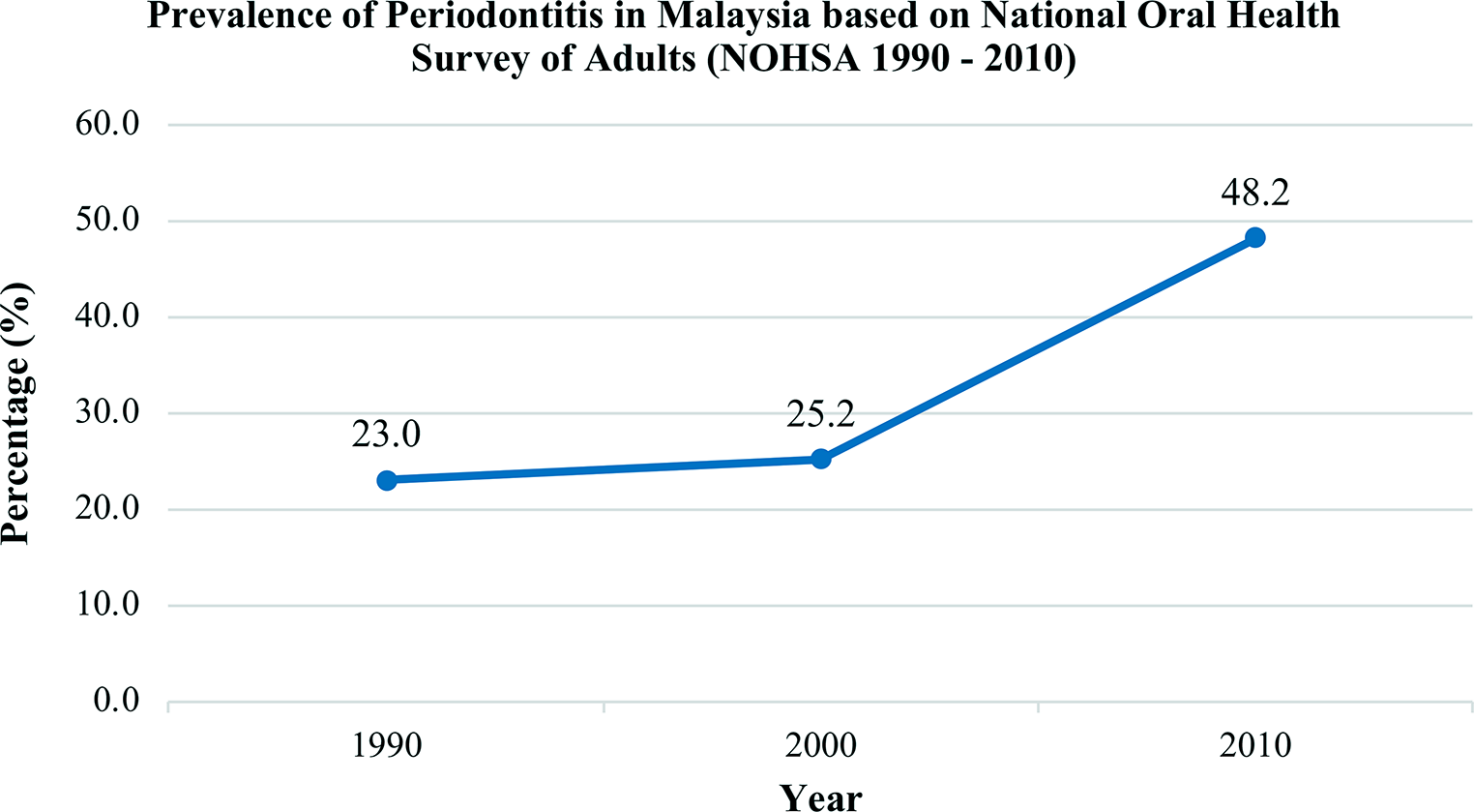
The trend of prevalence of periodontitis among adults in Malaysia (NOHSA 1990 - 2010)

Figure 1 shows the prevalence of periodontitis over three decades (1990–2010). Although the prevalence between 1990 and 2000 showed no significant increase, a substantial increase was observed between 2000 and 2010. Despite this, the prevalence of periodontitis was assumed to be unchanged or similar from 2010 to 2020 due to measures taken to control it. These measures included numerous oral health promotion initiatives and routine basic periodontal examination for all outpatients aged 16 years and above in public dental clinics. The effectiveness of these measures is yet to be evaluated. To determine the effectiveness of these measures in controlling the prevalence of periodontitis among adults in Malaysia between 2010 and 2020, small local studies were searched to obtain a more recent report on prevalence of periodontitis among adults in Malaysia. Four local studies published after 2010 were identified [13–16]. Despite systemic diseases being highly associated with periodontitis, the findings from these studies, which focused on populations with systemic diseases or health conditions, did not differ significantly from those reported in NOHSA 2010. This suggested that systemic conditions may not have a substantial influence on the prevalence of periodontitis. Therefore, these findings further support the argument that despite the association with systemic diseases, the prevalence of periodontitis did not change substantially.

To estimate the number of adults with periodontitis in Malaysia, the obtained proportion from the 2020 National Census [17] was multiplied with the 2010 NOHSA prevalence. The 2020 National Census is conducted once every 10 years in all residential areas throughout Malaysia. To check the robustness of the findings, one-way sensitivity analysis was conducted by varying the 2010 NOHSA prevalence of periodontitis using the reported 95% confidence interval [18].

### Estimating utilisation of oral healthcare services pattern among adults with periodontitis in Malaysia

In order to estimate the national economic burden of non-surgical periodontal management in specialist clinics in Malaysia, it is important to obtain information regarding the pattern of adults with periodontitis utilising oral healthcare services. Oral healthcare services in Malaysia are provisioned by both public and private sectors, however, there is no published study or report focusing on oral healthcare services utilisation among adults with periodontitis. The only available information on current utilisation of oral healthcare services was from the 2019 National Health and Morbidity Survey (NHMS) [19]. It was reported that among those who experienced recent oral health problems, only 23.0% sought dental treatment. This survey used to be conducted every ten years, but since 2011, the survey was conducted annually focusing on the scopes that need updating and recent health status for policy formulation. Thus, the information that best represent the utilisation of oral healthcare services among adults with periodontitis was the finding reported in this 2019 NHMS.

**Fig. 2:**
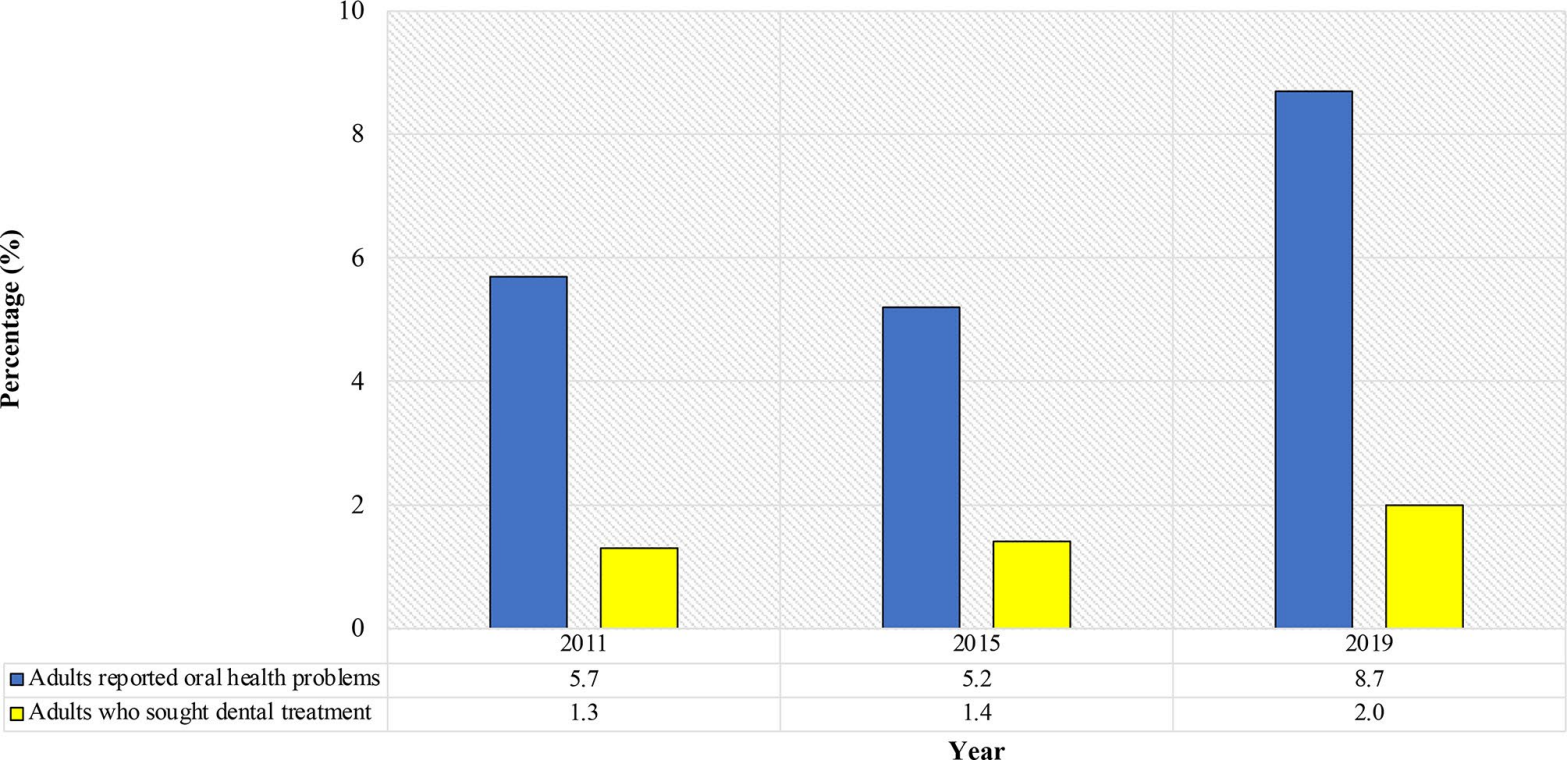
Trend of oral healthcare utilisation in Malaysia (NHMS 2011 - 2015)

To ensure that the prevalence of oral healthcare utilisation in 2019 NHMS can accurately estimate the utilisation in 2020, the trend of the utilisation was analysed (Fig. 2). Compared to 5.7% in 2011 and 5.2% in 2015, the proportion of adults experiencing recent oral health problems slightly increased to 8.7% in 2019. Regarding those who sought treatment for their oral health problems, there was a slight decrease from 26.6% (representing 1.3% of those who reported oral health problems) in 2015 to 23.0% (1.4% of those reported oral health problems) in 2019. However, the figure in 2019 was nearly similar to the figure reported in 2011 (21.9%), which constituted 2.0% of those who reported oral health problems [19]. Considering the small changes in both prevalence over eight years, it is unlikely for the prevalence in 2020 to differ much from the prevalence in 2019. Therefore, the used of the data from 2019 NHMS was deemed appropriate to estimate the utilisation of oral healthcare services among adults with periodontitis in Malaysia in 2020. Assuming that for an adult to report oral health problems, it must be associated with symptoms such as pain and mobility of tooth, the prevalence of adults with periodontitis who reported oral health problems and sought care in dental clinics was estimated using the following formula:

Number of adults reported oral health problems (a) = $\text{b} \text{x} \text{c}$

% of adults with periodontitis who reported oral health problems = $\frac{\text{d}}{(\text{d}+ \text{e})}$

Therefore, % of adults with periodontitis who reported oral health problem and sought treatment = $\text{a} \text{x} \left(\frac{\text{d}}{\text{d}+\text{e}}\right)\text{x} \text{f}$

with, b = prevalence of adults reported oral health problems.

c = number of adults (aged 15 years and above) in Malaysia in 2020.

d = prevalence of adults with severe periodontitis.

e = prevalence of adults with dental caries.

f = prevalence of adults reported oral health problems who sought treatment.

Then, using the annual mean number of visits per capita by sector, the proportion of the adults with periodontitis who sought care in both public and private dental clinics was estimated. Using the upper and lower limit of the 95% CI of all variables in the formula, the range of the number of adults with periodontitis who sought periodontal treatment was estimated. To check the robustness of the findings, one-way sensitivity analysis was performed using the estimated upper and lower range.

### Estimating direct medical costs of managing periodontitis in Malaysia

Information regarding the costs incurred by the oral healthcare providers for treating periodontitis in public and private sectors in Malaysia is not readily available. In this study, the direct medical costs of NSPT during the first year of periodontal management in specialist clinics from the oral healthcare provider’s perspective in both public and private facilities was estimated using multiple data sources. Firstly, the types of treatments provided based on patients’ periodontal status (stable, in remission, and unstable) were identified as shown in clinical pathway (Additional File 1). The pathway was developed in consultation with five periodontal specialists from both public (Ministry of Health Malaysia and Universiti Malaya) and private (Universiti Malaya Specialist Centre and private clinics) sectors specialising in periodontal treatment to ensure that the clinical pathway reflects the current NSPT in Malaysia. Then, the proportion of patients receiving the identified procedures was determined based on the periodontal specialists’ clinical experience and agreed by all periodontal experts.

This study used the Fees (Medical) (Cost of Services) Order 2014 as the source of information to determine the unit cost of each identified periodontal procedure in the public sector [20]. This document comprehensively describes the full cost of care of various medical and dental procedures. The set costs apply only to foreign person as they are expected to pay the full cost of care when they seek treatment in Malaysia. As for Malaysian, healthcare is highly subsidised. Therefore, the fee structure for citizens cannot be used as it does not reflect the actual cost of care. Thus, with the absence of readily available unit costs, the Fees (Medical) (Cost of Services) Order 2014 can be considered as the best available information that provides a close approximation of the actual unit costs of various periodontal treatment in public dental clinics. However, the cost for a few procedures was not found in the document, and these were obtained from previous local study on costs of periodontal treatment [21]. The unit cost for antibiotics was obtained from the Pharmaceutical Services Programme, Ministry of Health Malaysia Consumer Price Guide [22]. As for the private sector, the study assumed that OOP approximates the costs and profit. In Malaysia, the dental charges in private sectors are decided by free market forces. Thus, in this study, the fees charged by private dental providers working in the Universiti Malaya Specialist Centre was used as the base source. Additionally, two private dental practitioners specializing in periodontal treatment were interviewed to explore the range of periodontal treatment charges in private dental clinics.

To estimate the direct medical costs, we adjusted the unit costs of the identified procedures to MYR 2020 price using the Gross Domestic Product (GDP) deflator (base year varies by country) obtained from the world bank database [23]. The unit costs weighted by the proportion of patients receiving each procedure were added together to estimate the average direct medical costs. Subsequently, sensitivity analysis was conducted to ensure the robustness of findings by varying the average cost.

### Estimating the economic burden of non-surgical periodontal management in specialist clinics in Malaysia

To estimate the economic burden of non-surgical periodontal treatment in specialist clinics during the first year of periodontal management in Malaysia, we used a two-part analytical approach. Firstly, a decision analytical model was developed to outline the pre-determined treatment strategies and the possible consequences that could follow each strategy. Secondly, transitional probabilities between different periodontal health states following treatment were estimated using data from published network meta-analyses (NMA) [24]. The estimated direct medical costs and transitional probabilities were then used as inputs in the model to calculate the economic burden.

#### Structuring a decision model

**Fig. 3:**
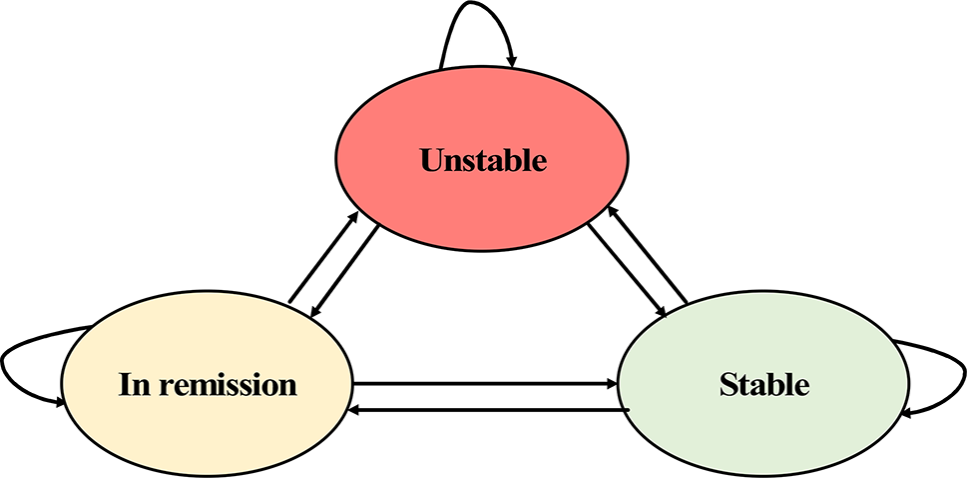
Markov model to estimate the economic burden of non-surgical periodontal management in specialist clinics in Malaysia

An irreducible type of Markov model (Fig. 3) was developed using Microsoft Excel (Version 16.61). This model was used considering the likelihood of a patient with periodontitis losing their teeth (absorbing state) within the first year of their periodontal treatment is low. Tooth loss can typically be prevented within four years after periodontal treatment [25]. Thus, patients will only transition to one of three periodontal health states throughout the whole model cycle. This model was developed based on the clinical pathway for managing periodontitis in consultation with relevant experts, including periodontists, health economists, and dental public health specialists.

#### Determining treatment strategies (base case)

Based on the periodontists’ feedback, the first year of periodontal treatment is mainly NSPT with or without the use of adjuncts. The commonly used adjuncts in the treatment of patients with periodontitis in Malaysia are a combination of amoxycillin (AMOX) and metronidazole (MET), as well as azithromycin (AZ). Additionally, based on their clinical experience, they estimated that only 20% of adults with periodontitis required the use of antibiotics, with 80% of them being prescribed AMOX and MET, and the remaining 20% being prescribed AZ. To date, there is no other adjuncts available in the public sector for the treatment of periodontitis in Malaysia. Other types of adjuncts available for periodontal procedures in the private sector are laser and antimicrobial photodynamic therapy but they are rarely used due to their high cost.

For this model, patients received one of the three treatment combination as they enter the model in unstable periodontal status. If the disease stabilises without recurrence at follow-up, patients are reviewed under supportive periodontal care. For patients in remission or unstable periodontitis, further treatment will be required in the form of re-instrumentation or periodontal surgery until the disease stabilises at follow-up. However, periodontal surgery was considered only in the fourth recall visit with the assumption that the likelihood of providing periodontal surgery during the first year of periodontal treatment is low. This is mainly because periodontal surgery can only be provided for patients with residual pocket of ≥ 5 mm despite having the ability to achieve and maintain self-performed oral hygiene and received adequate subgingival debridement. The model was evaluated with a cycle length of three months over a time horizon of one year.

#### Estimating the transition probabilities

As the length of each cycle of the model was three months, the transitional probabilities from stable to in remission, and unstable periodontal health state were estimated using data of short-term outcomes of the included studies in the NMA [24]. The method proposed by Hauri et al. [26] in combination with basic probability theory of two mutually exclusive events was used to determine the proportions of patients with stable, in remission, and unstable periodontal status. Next, the derived event proportion in both test and control group were pooled to obtain the relative risks (RR). Thereafter, the RR was used to derive transition probabilities following respective treatment strategies as suggested by Gidwani et al. [27]. As the data from the included studies in the NMA only allow estimation of transition probabilities from unstable to any other periodontal health states, a published study by Mdala et al. [28] was used to estimate the transition probabilities from stable or in remission to any other periodontal health states following periodontal treatment. The reported 1-year transitional probabilities were converted to the model cycle length of 3-months [27] and assumed to be fixed with respect to time.

#### Estimating the economic burden

To estimate the economic burden of non-surgical periodontal management in specialist clinics in Malaysia during the first year of management in 2020, direct medical costs incurred for non-surgical periodontal management in both public and private clinics, as well as transition probabilities from one periodontal status to another, were populated in the Markov model (Additional File 5). From the perspective of oral healthcare providers, the base case estimated the one-year economic burden of non-surgical periodontal management in specialist clinics in Malaysia in MYR 2020. To test the robustness of these estimates, one-way sensitivity analyses were conducted by varying input parameters over proportions, types of treatments, and costs, as per pre-determined scenarios formulated in consultation with health economy and periodontology experts from both public and private sectors. All calculations and data were performed using Microsoft Excel (Version 16.61).

## Results

### Clinical burden of adults with periodontitis in Malaysia

The number of adults estimated as having periodontitis in Malaysia was approximately 12 million (range: 11.4–12.6 million) (Table 1). Among them, about 7.5 million and 4.5 million were affected by the moderate and severe form the disease, respectively.

**Table 1:** Estimated number of adults with periodontitis in Malaysia in year 2020

	All			Moderate Periodontitis			Severe Periodontitis		
	Estimate	95% CI		Estimate	95% CI		Estimate	95% CI	
		Lower	Upper		Lower	Upper		Moderate (CPI 3)	Severe (CPI 4)
Prevalence of adults with periodontitis^1^	48.5%	46.0%	51.2%	30.3%	29.0%	31.6%	18.2%	17.0%	19.6%
Number of adults in Malaysia^2^	24,692,460								
Estimated number of adults with periodontitis	**11,975,843**	**11,358,532**	**12,642,540**	**7,481,815**	**7,160,813**	**7,802,817**	**4,494,028**	**4,197,718**	**4,839,722**

*Note *^1^Data obtained from 2010 NOHSA (OHD, 2013)

^2^Data obtained from 2020 National Census (DOSM, 2022)

### Utilisation of public and private oral healthcare services among adults with periodontitis in Malaysia

Only 8.7% of Malaysian adults reported recent oral health problems of which only 23% of them sought dental treatment. Of those who sought treatment, it was estimated that only 17% were affected by severe periodontitis (Table 2). The estimation showed that of 12 million adults with periodontitis, only 0.7% of them were aware of the disease and sought dental treatment. Whereas of adults with severe periodontitis, the estimated number of those who sought care was 1.9%. We also found that the annual mean number of visits in public and private sector 0.23 and 0.06, respectively, which indicates 79.3% of those who sought dental treatment utilised the public sector whereas 20.7% utilised the private sector. Thus, the estimated number of adults with periodontitis in Malaysia who sought dental treatment in public sector was 66,609. As for the private sector, periodontal treatment was sought by 17,387 adults with periodontitis (Table 3).

**Table 2:** Estimated number of adults reported oral health problems in the last 2 weeks and sought treatment in Malaysia in year 2020

	Reported oral health problems in the last 2 weeks^1^			Sought treatment^1^			Dental caries^2^			Severe periodontitis^2^		
	Estimate	95% CI		Estimate	95% CI		Estimate	95% CI		Estimate	95% CI	
		Lower	Upper		Lower	Upper		Lower	Upper		Lower	Upper
Proportion of adults	8.7%	7.9%	9.5%	23.0%	20.1%	26.2%	83.0%	83.8%	82.1%	17.0%	16.2%	17.9%
Number of adults in Malaysia^2^	24,692,460			2,148,244	1,950,704	2,345,784	494,096	392,092	614,595	494,096	392,092	614,595
Estimated number of adults	**2,148,244**	**1,950,704**	**2,345,784**	**494,096**	**392,092**	**614,595**	**410,100**	**328,573**	**504,582**	**83,996**	**63,519**	**110,013**

*Note *^1^Data obtained from 2019 NHMS (IHSR, 2020)

^2^Data obtained from 2020 National Census (DOSM, 2022)

**Table 3:** Estimated number of adults with periodontitis reported oral health problems in the last 2 weeks and sought treatment by sectors in Malaysia in year 2020

Sector of the oral healthcare facilities	Public			Private		
	Estimate	95% CI		Estimate	95% CI	
		Lower	Upper		Lower	Upper
Annual mean number of visits to oral healthcare facilities per capita by sector^1^	0.23	0.22	0.25	0.06	0.05	0.07
Composition of adults with periodontitis reported oral health problems in the last 2 weeks and sought treatment by sectors (%)	79.3%	81.5%	78.1%	20.7%	18.5%	21.9%
Number of adults (aged ≥15 years) with periodontitis who sought treatment^2^	83,996	63,519	110,013	83,996	63,519	110,013
Estimated number of adults with periodontitis who received dental treatment	**66,609**	**51,768**	**85,920**	**17,387**	**11,751**	**24,093**

*Note *^1^Data obtained from 2019 NHMS (IHSR, 2020)

^2^Estimated number of adults with periodontitis who utilised oral healthcare services in Table 2

### Direct medical costs of different non-surgical periodontal treatment modalities in Malaysia

**Fig. 4:**
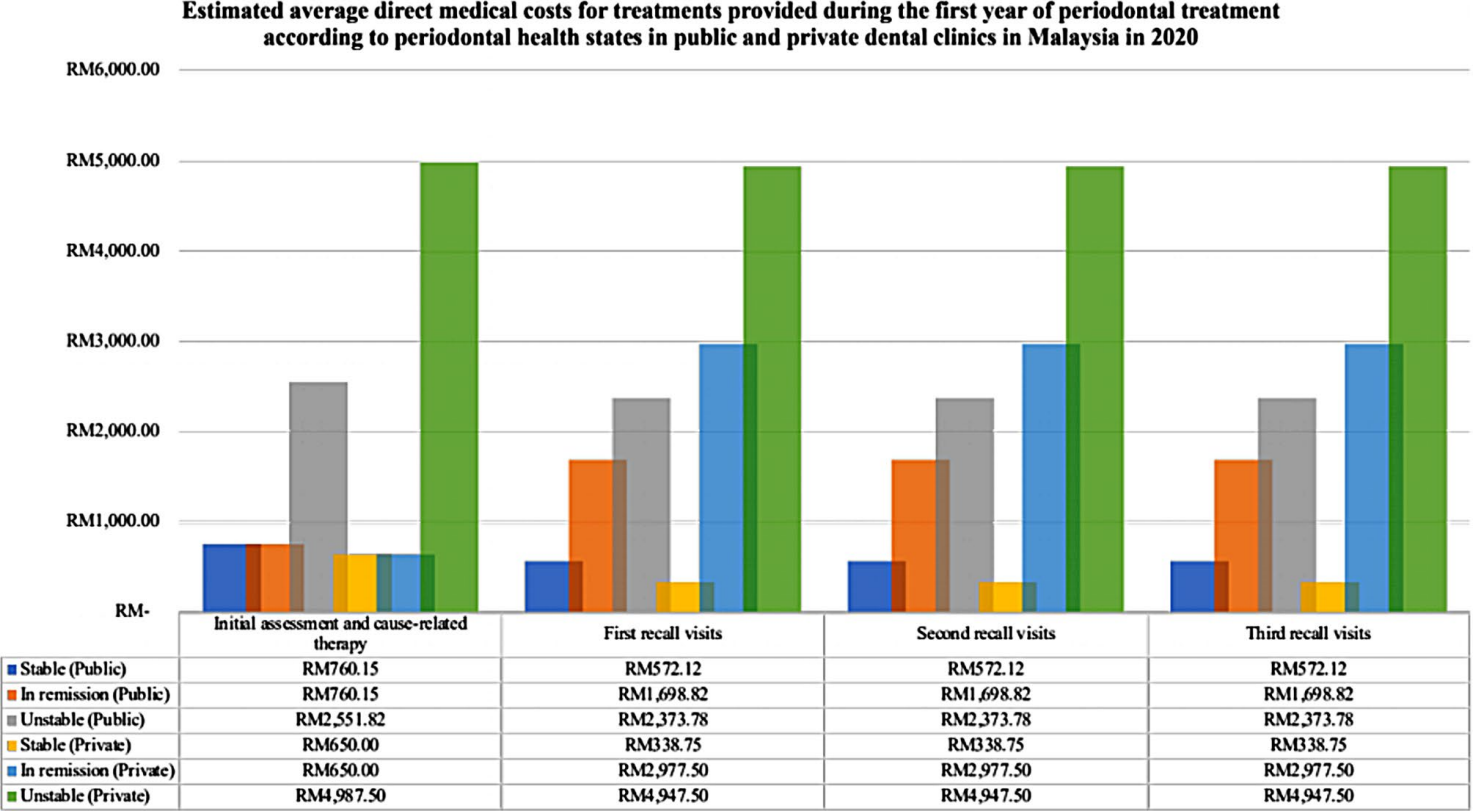
The estimated direct medical costs for non-surgical periodontal treatment during the first year of periodontal management according to periodontal health states

**Fig. 5:**
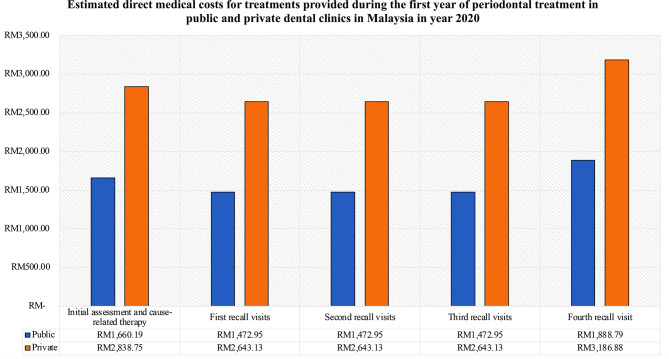
The estimated direct medical costs for non-surgical periodontal treatment during the first year of periodontal management in public and private dental clinics

The details of the periodontal treatment provided along with its unit cost and proportions of patients are presented in Supplementary Material 1.  With regards to the average direct costs of treating patients with different periodontal health state, higher costs incurred when treating unstable patients as compared with patients in stable and in remission periodontal health state (Fig. 4). In terms of the costs in different sectors, the average costs of periodontal treatment in the public dental clinics were about half of the private direct costs at every follow-up periods. The estimated average medical costs at every follow-up in both public and private are illustrated in Fig. 5.

Tables 4 and 5 summarise the average direct costs of treating patients with periodontitis in public and private dental clinics, respectively. Based on the sensitivity analysis, the range of estimated direct medical costs during the initial assessment and cause-related therapy in public clinics was MYR 760–3,072 (≈USD 181–732), whereas private dental clinics was MYR 650–8,105/patient (≈USD 155–1,931/patient). Meanwhile, the costs inflicted during the following three recall visits were estimated between MYR 458–2,849/patient (≈USD 109–679/patient) in public and MYR 128–8,100/patient (≈USD 3 − 1,929/patient) in private clinics. During the fourth recall visit, the direct medical costs can be from MYR 500 (≈USD 119) to MYR 3,783 (≈USD 901) per patient in public facilities, and MYR 227 (≈USD 54) up to MYR 9,600 (≈USD 2,287) per patient in private facilities.

**Table 4:** Estimated direct medical costs for non-surgical periodontal treatment during the first year of periodontal management in public dental clinics in year 2020

Visits	Costs according to periodontal health states (in 2020 MYR/patient)								
	Stable^1^			In remission^2^			Unstable^3^		
	Average	-20%	+ 20%	Average	-20%	+ 20%	Average	-20%	+ 20%
**Public**									
**Initial assessment + cause-related therapy**									
• NSPT only	760.15	608.12	912.18	760.15	608.12	912.18	2,551.82	2,041.46	3,062.18
• NSPT + AMOX + MET	768.55	614.84	922.26	768.55	614.84	922.26	2,560.22	2,048.18	3,072.26
• NSPT + AZ	767.05	613.64	920.46	767.05	613.64	920.46	2,558.72	2,046.98	3,070.46
**First recall visit**	572.12	457.70	686.54	1,698.82	1,359.06	2,038.58	2,373.78	1,899.02	2,848.54
**Second recall visit**	572.12	457.70	686.54	1,698.82	1,359.06	2,038.58	2,373.78	1,899.02	2,848.54
**Third recall visit**	572.12	457.70	686.54	1,698.82	1,359.06	2,038.58	2,373.78	1,899.02	2,848.54
**Fourth recall visit**	625.46	500.37	750.55	1,698.82	1,359.06	2,038.58	3,152.12	2,521.70	3,782.54

^1^Patients with PPD 4 mm and less, no BOP at 4 mm sites, BOP < 10%

^2^Patients with PPD 4 mm and less with BOP, BOP > 10%

^3^Patients with PPD 5 mm and more

±20% represents the varying range used in the sensitivity analysis for cost estimation

**Table 5:** Estimated direct medical costs for non-surgical periodontal treatment during the first year of periodontal management in private dental clinics in year 2020

Visits	Costs according to periodontal health states (in 2020 MYR/patient)								
	Stable			In remission			Unstable		
	Average	95% CI		Average	95% CI		Average	95% CI	
		**Lower**	**Upper**		**Lower**	**Upper**		**Lower**	**Upper**
**Private**									
**Initial assessment + cause-related therapy**									
• NSPT only	650.00	500.00	900.00	650.00	500.00	900.00	4,987.50	2,000.00	8,075.00
• NSPT + AMOX + MET	690.00	530.00	950.00	690.00	530.00	950.00	5,027.50	2,030.00	8,105.00
• NSPT + AZ	690.00	530.00	950.00	690.00	530.00	950.00	5,027.50	2,030.00	8,105.00
**First recall visit**	338.75	127.50	550.00	2,977.50	1,155.00	4,800.00	4,947.50	1,795.00	8,100.00
**Second recall visit**	338.75	127.50	550.00	2,977.50	1,155.00	4,800.00	4,947.50	1,795.00	8,100.00
**Third recall visit**	338.75	127.50	550.00	2,977.50	1,155.00	4,800.00	4,947.50	1,795.00	8,100.00
**Fourth recall visit**	488.75	227.50	750.00	2,977.50	1,155.00	4,800.00	5,885.00	2,170.00	9,600.00

^1^Patients with PPD 4 mm and less, no BOP at 4 mm sites, BOP < 10%

^2^Patients with PPD 4 mm and less with BOP, BOP > 10%

^3^Patients with PPD 5 mm and more

### National economic burden of non-surgical periodontal management in specialist dental clinics

Based on the base case analysis, the estimated national economic burden of non-surgical periodontal management in both public and private specialist clinics in Malaysia in 2020 was approximately MYR 696 million (≈ USD 166 million; average exchange rate of 0.2382). Thus, approximately MYR 8,283 (≈USD 1,9773) per patient per year was required for non-surgical periodontal management in specialist clinics in Malaysia, in which about MYR 7,277 (≈USD 1,733) and MYR 12,139 (≈USD 2,892) per patient per year was incurred in public and private sector, respectively. The estimated national economic burden is presented in Table 6. The results of sensitivity analyses showed that variations in the treatment strategies, have almost no influence on the base-case estimates (Table 7). When alterations were done using the lower limit of proportions of adults with periodontitis who sought dental treatment, only slight reduction compared with the base-case estimate was observed. However, when upper limits of the both proportion and direct medical costs, as well as the lower limit of the direct medical cost were applied to the model, the base-case estimates changed by approximately 30%. Based on this analysis, it was determined that the one-year national economic burden of non-surgical periodontal management in specialist clinics in Malaysia ranged between MYR 471 million (≈USD 112 million) to MYR 922 million (≈USD 220 million) in year 2020.

**Table 6:** Economic burden of non-surgical periodontal management in specialist clinics in Malaysia in year 2020

	No. of treated adults with periodontitis	Direct medical costs	
		MYR 2020	USD 2020
Public dental clinics	66,609	484,733,861.59	115,463,605.83
Private dental clinics	17,387	211,065,050.54	50,275,695.04
**Economic burden**	**83,996**	**695,798,912.13**	**165,739,300.87**

**Table 7:** Sensitivity analyses of the economic burden estimates of non-surgical periodontal treatment during the first year of periodontal management in specialist clinics in Malaysia in year 2020

	Settings	No. of treated adults with periodontitis	Direct medical costs (MYR 2020)	Economic burden (MYR 2020)	Difference compared with base-case estimate
Varied parameters	**(a) Exclude the use of adjuncts**				
	Public	66,609	486,009,658.28	698,017,987.11	+ 0.32%
	Private	17,387	212,008,328.83		
	Private	17,387	213,030,476.87		
	**(b) Lower limit of the proportion of adults with periodontitis who received dental treatment**				
	Public	51,768	484,770,729.63	627,418,852.89	− 9.83%
	Private	11,751	142,648,123.26		
	**(c) Upper limit of the proportion of adults with periodontitis who received dental treatment**				
	Public	85,920	625,275,078.80	917,745,300.22	+ 31.90%
	Private	24,093	292,470,221.42		
	**(d) Lower limit of the treatment fee**				
	Public	66,609	387,788,229.98	470,834,826.64	− 32.33%
	Private	17,387	83,046,596.66		
	**(e) Upper limit of the treatment fee**				
	Public	66,609	581,679,493.21	922,432,157.63	+ 32.57%
	Private	17,387	340,752,664.42		

## Discussion

Periodontitis, sharing common risk factors and social determinants with major NCDs that are responsible for two-thirds of deaths worldwide, like cardiovascular disease (CVD), diabetes, and cancer [9, 29]. In Malaysia, diabetes accounted for MYR 4.38 billion (≈USD1.02 billion; average exchange rate of 0.2328; 45.38%), followed by CVD with MYR 3.93 billion (≈USD 0.91 billion; 40.73%), and cancer with MYR 1.34 billion (≈USD 0.30 billion; 13.89%) of the total costs of NCDs in 2017 [30]. Despite the low prevalence of patients receiving periodontal treatment in specialist clinics in Malaysia, the economic burden of non-surgical periodontal management was estimated to be equal to one-sixth of the cost of NCDs. This highlights the urgent need for effective policies and strategies to combat periodontitis among adults in Malaysia, a disease that can largely be prevented with adequate oral hygiene practice.

Prior to this study, there was only one local study that estimated the costs of providing treatment for adults with periodontitis in public sector specialist dental clinics in Malaysia [31]. However, the cost estimation in the study was from both public provider and societal perspectives and did not include private dental clinics. In contrast, the present study estimated costs from the oral healthcare provider’s perspective from both public and private oral health facilities. This was done to accurately reflect the Malaysian dual healthcare system where oral healthcare is provided by both public and private sectors. The public sector is heavily funded by general taxation with minimum user fees, whilst the private sector is largely funded by OOP. The estimation of costs for public clinics was about three times higher than the previous study, likely due to the differences in methodology.

The present study estimated costs based on patients’ periodontal health states and weighted by the proportion of patients receiving identified procedures. This approach was used to accurately cost a range of procedures with various uncertainties with regards to treatment modalities and patient response at every visit. The direct medical costs estimated for non-surgical periodontal management in specialist clinics in Malaysia were MYR 8,863 (≈USD 2,111) per patient per year in public and private dental clinics. The previous study’s estimated average cost of MYR 2,764 (≈USD 658) per patient per year for public clinics may be an underestimate considering the implementation of the current classification of periodontitis and its associated management. It is also important to note that the determination of dental fees in private clinics is influenced by free market forces, resulting in a wide range of costs for care. Thus, this study provides a more comprehensive estimate of the direct medical costs of treating periodontitis in Malaysia from the oral healthcare provider’s perspective and highlights the differences in costs between public and private dental clinics.

The previous study by Mohd-Dom et al. estimated the economic burden of periodontitis in Malaysia in 2012 to be MYR 32.5 billion (≈USD 10.53 billion; average exchange rate of 0.3240) to society and MYR 27.1 billion (≈USD 8.75 billion) to providers [32]. The economic burden to providers in the present study is lower by 67.2%, possibly due to the different estimation methods. The previous study considered all adults with periodontitis, whilst the present study only considered adults with periodontitis who sought dental treatment. This approach is necessary because only 27.4% of adults in Malaysia utilised oral healthcare services in the last year, and periodontitis often goes undetected until it reaches a late stage, resulting in higher healthcare costs. By focusing on adults who are aware of their periodontal condition and seek treatment, this study accurately reflects the current situation of managing adults with periodontitis and estimates the economic burden more accurately. It is also worth noting that the previous study only estimated the economic burden from the perspective of the Ministry of Health as the provider. However, the oral healthcare system in Malaysia follows a dual healthcare system that comprises both public and private sectors [7]. Therefore, to obtain a more comprehensive estimation of the economic burden of non-surgical periodontal management, this present study included both public and private oral healthcare providers.

While previous study on economic burden of periodontitis considered both direct and indirect costs from the societal perspective [32], this study focused solely on direct medical costs were included. This decision was based on availability reliable data [20–22] and inputs from experienced periodontal specialists, ensuring a credible reflection of the national economic burden of periodontitis to oral healthcare providers in Malaysia. Additionally, the previous study included rehabilitative dental procedures in their estimation [32], but such procedures were not included in this study as they must be a sequela to periodontitis to be included in the economic burden estimation and unlikely to be provided during the first year of periodontal treatment.

The study found that MYR 180 million (≈USD 42.88 million) was spent on direct medical costs for treating periodontitis in private dental clinics, which is a significant finding. However, as private health insurance does not cover most dental care, private dental care in Malaysia is paid from OOP, which is the least progressive form of health financing. This means that the burden of these costs falls disproportionately on vulnerable groups, as OOP payments can be welfare reducing and can lead to financial hardship [33, 34]. While previous local study has shown that OOP payments for healthcare in Malaysia are pro-rich [35], it is unclear how this distribution applies specifically to dental care. To address this issue, it is important to establish stewardship in oral healthcare and to provide quality and efficient oral healthcare services that reduce OOP costs.

The study by Mohd-Dom et al. was the first to estimate the economic burden of periodontal disease in Malaysia, demonstrating the crucial role of economic evidence in decision-making [32]. While the Ministry of Health had already taken action to address the high prevalence of periodontal disease following the 2010 NOHSA [1], the estimates provided by the authors emphasised the need for continued efforts to tackle this disease. This current study builds on the previous work by providing an updated economic framework for estimating the burden of periodontitis in Malaysia, utilising an irreducible Markov model. Importantly, this study is the first to use a decision analytical model to estimate the economic burden of oral diseases in Malaysia, marking a significant advancement in the field. The utilisation of the Markov model to estimate the economic burden of periodontitis in Malaysia was driven by the complexity and unpredictability associated with the treatment of this disease [36]. A systematic approach was required to make informed decisions despite these uncertainties. By utilising a mathematical approach, the model was able to evaluate all possible clinical outcomes resulting from a set of interventions, in addition to the costs of care. Through the incorporation of transition probabilities for each consequence, the likelihood of each outcome could be estimated. Furthermore, each outcome had a cost associated with it. Thus, the expected cost and expected outcome of each option could be determined based on the inputs into the model.

An important methodological consideration in this study was the decision to limit the Markov model to four cycles, representing a one-year timeframe. This approach aligned with the availability and reliability of current data, which was more conducive to short-term modelling. Longer-term models would require extensive, longitudinal data, potentially introducing greater uncertainty over prolonged periods [36]. Additionally, the one-year cycle allowed for a focused analysis of the immediate progression and treatment responses of periodontal disease, essential for understanding short-term impacts and costs. This timeframe also ensured that the findings remained relevant and adaptable to the rapidly changing healthcare policies, technological advancements, and treatment practices. Efficient and timely resource allocation was facilitated, reflecting the typical annual budgeting processes in healthcare settings. While serving as a foundational baseline for future research, this one-year model also sets the stage for future expansions to explore longer-term scenarios, providing a comprehensive view of the economic burden of periodontal management. This approach, capturing the immediate economic impact, paves the way for future research to expand upon these findings and explore the long-term economic implications of periodontal disease management.

Being the first study to use a decision analytic model to estimate the economic burden of non-surgical periodontal management in specialist clinics, the framework used in this study can be adapted to estimate the economic burden of periodontitis in other countries. Furthermore, the model can also serve as a reference to estimate the economic burden of other oral diseases. The study findings highlight the significant economic impact of periodontitis treatment in Malaysia, particularly in the public sector. While individual-level prevention efforts remain crucial, this study provides valuable economic arguments for advocating for periodontal disease prevention and control at the community and national levels. Currently, there is a lack of public health measures aimed at preventing and controlling periodontitis [37]. The implementation of public health measures could potentially be a cost-effective approach, as it could help prevent tooth loss and other adverse consequences related to oral health among patients [38]. Therefore, it is time to consider implementing core public health activities, including assessment, policy development, and assurance. The economic burden estimates in this study can facilitate policy development by demonstrating the impact of periodontal disease and emphasising its importance as a major public health problem and NCD.

Transitioning from a focus on public health strategies, it is also relevant to consider the role of university hospitals and specialist clinics associated with private higher education institutions in this context. These entities, both in the public and private sectors, play a supportive role in the broader healthcare landscape, potentially mitigating the burden of periodontal disease management. Their involvement in providing specialised care and facilitating capacity building in periodontal treatment offers a valuable dimension to the overall strategy for addressing periodontal health in Malaysia. While the primary focus of our research was on the direct costs associated with specialist clinics, the integration of university hospitals and private higher education institutions-affiliated clinics into the NSPT framework could contribute to a more balanced distribution of care and potentially alleviate some of the economic pressures identified in this study. This aspect, though not the central theme of this research, highlights the importance of a multi-faceted approach in periodontal disease management and warrants attention in future studies.

However, in addressing the economic burden of non-surgical periodontal management in Malaysia, this study presents findings that are inherently specific to the Malaysian healthcare context. It is important to note that direct extrapolation of these economic outcomes to other countries is not feasible due to the distinct nature of healthcare systems, economic conditions, and cost structures in different regions. Nevertheless, the methodology and framework utilised in this study offer a valuable model for conducting similar economic analyses in diverse healthcare settings. While the cost estimations are presented in both MYR and USD to aid comprehension and comparison for an international audience, caution is advised against a simplistic interpretation of these figures across different national boundaries. The findings of this study have the potential to inform global strategies in periodontal disease management by providing a template for economic analyses and emphasising the importance of context-specific approaches. It sheds light on the economic burden of periodontal disease in Malaysia and sets the stage for future research to build upon these findings, exploring broader applications and implications in the field of oral healthcare. Researchers and policymakers in other countries are encouraged to adapt the foundational approach of this study, tailoring it to their unique healthcare environments to estimate the economic impact of managing periodontal diseases. Such tailored analyses are crucial for developing effective, context-specific strategies for periodontal disease management worldwide.

## Conclusion

The findings of this study demonstrate substantial economic burden of non-surgical periodontal management in specialist clinics in Malaysia. Being a life-long disease, these findings highlight the importance of enforcing primary and secondary preventive measures. Furthermore, on the strength and reliability of this economic evidence, this study provides vital information to inform policy- and decision-making regarding the future direction of managing of periodontitis in Malaysia.

### Electronic supplementary material

Below is the link to the electronic supplementary material.

**Supplementary Material 1**: Additional files for estimating the economic burden of non-surgical periodontal management in Malaysian Specialist Clinics.

## Data Availability

The data used and/or analysed in this study are available from the corresponding author on reasonable request.

## Abbreviations

AMOX: amoxycillin
AZ: azithromycin
CVD: cardiovascular disease
GDP: Gross Domestic Product
MET: metronidazole
NCDs: non-communicable diseases
NMA: network meta-analysis
NSPT: non-surgical periodontal treatment
NHMS: National Health and Morbidity Survey
NOHSA: National Oral Health Survey for Adults
OOP: out-of-pocket payment
RR: relative risk
