# Supplementary material for: Modelling the national economic burden of non-surgical periodontal management in specialist clinics in Malaysia using a markov model
Source: BMC Oral Health. 2024 Mar 18;24:346. doi: 10.1186/s12903-024-04094-z (PMC10949624; doi:10.1186/s12903-024-04094-z)
Supplement: Supplementary file 1 — Supplementary Material 1: Additional files for estimating the economic burden of non-surgical periodontal management in Malaysian Specialist Clinics. [file 12903_2024_4094_MOESM1_ESM.docx]

**Additional File 1 -** Clinical pathway of managing patients with periodontitis.

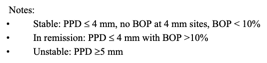


**Additional File 2 -** Estimated average direct medical costs for treatments provided during the first year of periodontal treatment according to periodontal health states in public dental clinics.

| **No.** | **Treatments** | **Unit cost (in 2020 MYR)** | **Treatment proportion among patients with periodontitis** | | | **Source** |
| --- | --- | --- | --- | --- | --- | --- |
|  |  |  | **Stable** | **In remission** | **Unstable** |  |
| 1. | **Initial assessment and cause-related therapy** | | | | | |
|  | Specialist outpatient | 128.03 | 100% | 100% | 100% | a (Page 227) |
|  | Full mouth periodontal assessment | 99.66 | 100% | 100% | 100% | b |
|  | Electric pulp test | 76.66 | 10% | 10% | 50% | b |
|  | Orthopantomograph | 74.69 | 0% | 0% | 100% | a (Page 310) |
|  | Periapical radiograph | 896.24 | 50% | 50% | 100% | a (Page 310) |
|  | Oral hygiene instruction | 76.66 | 100% | 100% | 100% | b |
|  | Subgingival debridement (full mouth) | 679.65 | 0% | 0% | 50% | a (Page 250) |
|  | Subgingival debridement (per quadrant) | 1796.75 | 0% | 0% | 50% | a (Page 250) |
|  | **Treatment costs** |  | **760.15** | **760.15** | **2551.82** |  |
| 2. | **First recall visit** |  |  |  |  |  |
|  | Specialist outpatient | 128.03 | 100% | 100% | 100% | a (Page 227) |
|  | Periodontal reassessment | 132.52 | 100% | 100% | 100% | b |
|  | Oral hygiene reinforcement | 83.23 | 100% | 100% | 100% | b |
|  | Scaling and polishing as indicated | 456.66 | 50% | 100% | 100% | a (Page 246) |
|  | Subgingival debridement (full mouth) | 679.65 | 0% | 0% | 20% | a (Page 250) |
|  | Subgingival debridement (per quadrant) | 1796.75 | 0% | 50% | 80% | a (Page 250) |
|  | **Treatment costs** |  | **572.12** | **1698.82** | **2373.78** |  |
| 3. | **Second recall visit** |  |  |  |  |  |
|  | Specialist outpatient | 128.03 | 100% | 100% | 100% | a (Page 227) |
|  | Periodontal reassessment | 132.52 | 100% | 100% | 100% | b |
|  | Oral hygiene reinforcement | 83.23 | 100% | 100% | 100% | b |
|  | Scaling and polishing as indicated | 456.66 | 50% | 100% | 100% | a (Page 246) |
|  | Subgingival debridement (full mouth) | 679.65 | 0% | 0% | 20% | a (Page 250) |
|  | Subgingival debridement (per quadrant) | 1796.75 | 0% | 50% | 80% | a (Page 250) |
|  | **Treatment costs** |  | **572.12** | **1698.82** | **2373.78** |  |
| 4. | **Third recall visit** |  |  |  |  |  |
|  | Specialist outpatient | 128.03 | 100% | 100% | 100% | a (Page 227) |
|  | Periodontal reassessment | 132.52 | 100% | 100% | 100% | b |
|  | Oral hygiene reinforcement | 83.23 | 100% | 100% | 100% | b |
|  | Scaling and polishing as indicated | 456.66 | 50% | 100% | 100% | a (Page 246) |
|  | Subgingival debridement (full mouth) | 679.65 | 0% | 0% | 20% | a (Page 250) |
|  | Subgingival debridement (per quadrant) | 1796.75 | 0% | 50% | 80% | a (Page 250) |
|  | **Treatment costs** |  | **572.12** | **1698.82** | **2373.78** |  |
| 5. | **Fourth recall visit** |  |  |  |  |  |
|  | Specialist outpatient | 128.03 | 100% | 100% | 100% | a (Page 227) |
|  | Periodontal reassessment | 132.52 | 100% | 100% | 100% | b |
|  | Fluoride varnish application | 106.70 | 50% | 0% | 0% | a (Page 248) |
|  | Oral hygiene reinforcement | 83.23 | 100% | 100% | 100% | b |
|  | Scaling and polishing as indicated | 456.66 | 50% | 100% | 100% | a (Page 246) |
|  | Subgingival debridement (full mouth) | 679.65 | 0% | 0% | 20% | a (Page 250) |
|  | Subgingival debridement (per quadrant) | 1796.75 | 0% | 50% | 80% | a (Page 250) |
|  | Periodontal surgery | 1556.69 | 0% | 0% | 50% | a (Page 389) |
|  | **Treatment costs** |  | **625.46** | **1698.82** | **3152.12** |  |

1. Fees (Medical) (Cost of Services) Order 2014 (P.U.(A) 363/2014)
2. Local study by Mohd-Dom et al. (2016)

| **Medication costs:**  AMOX+MET: MYR 8.40 (Consumer Price Guide MOH)  AZ: MYR6.90 (Consumer Price Guide MOH) | **Adjustment to 2020 prices**  2012 GDP deflator 96.24960076  2014 GDP deflator 98.79660232  2020 GDP deflator 105.4115695  Source: https://databank.worldbank.org/source/world-development-indicators# |
| --- | --- |

**Additional File 3** - Estimated average direct medical costs for treatments provided during the first year of periodontal treatment according to periodontal health states in private dental clinics.

| **No.** | **Treatments** | **Unit cost (in MYR 2020)** | **Treatment proportion among patients with periodontitis** | | | **Source** |
| --- | --- | --- | --- | --- | --- | --- |
|  |  |  | **Stable** | **In remission** | **Unstable** |  |
| 1. | **Initial assessment and cause-related therapy** |  |  |  |  |  |
|  | Consultation + Examination + Treatment plan | 200.00 | 100% | 100% | 100% | a & b |
|  | Orthopantomograph | 212.50 | 0% | 0% | 100% | a & b |
|  | Periapical radiograph | 900.00 | 50% | 50% | 100% | a & b |
|  | Subgingival debridement (Full mouth) | 2350.00 | 0% | 0% | 50% | b |
|  | Subgingival debridement (per quadrant) | 5000.00 | 0% | 0% | 50% | a |
|  | **Treatment costs** |  | **650.00** | **650.00** | **4987.50** |  |
| 2. | **First recall visit** |  |  |  |  |  |
|  | Consultation + Examination + Treatment plan | 200.00 | 100% | 100% | 100% | a & b |
|  | Subgingival debridement (Full mouth) | 2350.00 | 0% | 0% | 20% | b |
|  | Subgingival debridement (per quadrant) | 5000.00 | 0% | 50% | 80% | a |
|  | Scaling and polishing as indicated | 277.50 | 50% | 100% | 100% | a & b |
|  | **Treatment costs** |  | **338.75** | **2977.50** | **4947.50** |  |
| 3. | **Second recall visit** |  |  |  |  |  |
|  | Consultation + Examination + Treatment plan | 200.00 | 100% | 100% | 100% | a & b |
|  | Subgingival debridement (Full mouth) | 2350.00 | 0% | 0% | 20% | b |
|  | Subgingival debridement (per quadrant) | 5000.00 | 0% | 50% | 80% | a |
|  | Scaling and polishing as indicated | 277.50 | 50% | 100% | 100% | a & b |
|  | **Treatment costs** |  | **338.75** | **2977.50** | **4947.50** |  |
| 4. | **Third recall visit** |  |  |  |  |  |
|  | Consultation + Examination + Treatment plan | 200.00 | 100% | 100% | 100% | a & b |
|  | Subgingival debridement (Full mouth) | 2350.00 | 0% | 0% | 20% | b |
|  | Subgingival debridement (per quadrant) | 5000.00 | 0% | 50% | 80% | a |
|  | Scaling as indicated | 277.50 | 50% | 100% | 100% | a & b |
|  | **Treatment costs** |  | **338.75** | **2977.50** | **4947.50** |  |
| 5. | **Fourth recall visit** |  |  |  |  |  |
|  | Consultation + Examination + Treatment plan | 200.00 | 0% | 100% | 100% | a & b |
|  | Maintenance phase | 350.00 | 100% | 0% | 0% | a |
|  | Subgingival debridement (Full mouth) | 2350.00 | 0% | 0% | 20% | b |
|  | Subgingival debridement (per quadrant) | 5000.00 | 0% | 50% | 80% | a |
|  | Scaling and polishing as indicated | 277.50 | 50% | 100% | 100% | a & b |
|  | Periodontal surgery | 1875.00 | 0% | 0% | 50% | a & b |
|  | **Treatment costs** |  | **488.75** | **2977.50** | **5885.00** |  |

1. UMCS Fee schedule
2. Interview with private practitioners

| **Medication costs:**  AMOX+MET: MYR40.00/complete dosage  AZ: MYR40.00/complete dosage | **Adjustment to 2020 prices**  2012 GDP deflator 96.24960076  2014 GDP deflator 98.79660232  2020 GDP deflator 105.4115695  Source: https://databank.worldbank.org/source/world-development-indicators# |
| --- | --- |

**Additional 4 –** Equation used to estimate transition probabilities.

The method proposed by Hauri et al. (2008) was used to determine the proportions of PPD ≥ 5 mm from the available data sets (Equation 1).

**Equation 1** Determining the proportions of event in treatment group.

| P (X ≤ x) = F (X)(x) = φ ((x-μ) / σ) |
| --- |

Proportion of patients with in-remission periodontal health states (i.e., PPD ≤ 4 mm with BOP >10%) cannot be estimated directly using method proposed by Hauri et al. (2008). Therefore, prior to estimating the proportion of PPD ≤ 4 mm with BOP >10%, modification was made to obtain the probability of two mutually exclusive events (i.e., PPD ≤ 4 mm and BOP >10%) happening together using the following equation of basic probability theory, where P(A) is the probability of event A and P (B) is the probability event B as described Equation 2. Subsequently, the obtained probability was used to estimate the proportion.

**Equation 2** Calculating the probability of two events happening together.

| P(A∩B) = P(A) x P(B) |
| --- |

Next, the probability of PPD ≤ 4 mm was calculated by subtracting total probability of PPD ≥ 5 mm and PPD ≤ 4 mm with BOP >10% from one (Equation 3). This is because the sum of the probabilities of all the possible values is one. Then, the proportion of patients with PPD ≤ 4 mm were estimated using the obtained probability.

**Equation 3** Obtaining probability of an events from sum of the probabilities.

| P(C) = 1 - P(A) - P(B) |
| --- |

Next, the derived event proportion in both test and control group were pooled to obtain the relative risks (RR). Thereafter, the RR was used to derive transition probabilities following respective treatment strategies. Equation 4 was used to estimate the transition probabilities, where *p_1_* is the probability of the events in treated patients, and *p_0_* is the probability of the events in untreated patients.

**Equation 4** Deriving transitional probabilities from relative risk of treated group.

| $RR=(p1/p0 )$  $p1=RR x p0=(p1/p0 ) x p0$ |
| --- |

As the data from the included studies in the NMA only allow estimation of transition probabilities from unstable to any other periodontal health states, another source is required to inform the transition probabilities from stable or in remission to any other periodontal health states. Through literature search, a published study by Mdala et al. (2014) that estimated the transition probabilities from stable or in remission to any other periodontal health states following periodontal treatment was identified. The reported 1-year transitional probabilities reported in Mdala et al. (2014) was then converted to the model cycle length of 3-months using Equation 5. These transition probabilities of periodontal health states were used as input in the Markov model to estimate the economic burden of managing periodontitis in Malaysia and assumed to be fixed with respect to time.

**Equation 5** Converting transition probabilities to the model’s cycle length.

| $p={1-\left( 1-12 months transition probability \right)}^{\frac{1}{4}}$ |
| --- |

**Additional File 5 -** Transition probabilities from one periodontal health state to another

| **State at start of cycle** | **State at end of cycle** | | |
| --- | --- | --- | --- |
| 1. **NSPT only** | | | |
|  | **Stable** | **In remission** | **Unstable** |
| **Stable^1^** | 0.966 | 0.031 | 0.003 |
| **In remission^1^** | 0.331 | 0.661 | 0.008 |
| **Unstable^2^** | 0.215 | 0.615 | 0.170 |
| 1. **NSPT + AMOX + MET** | | | |
|  | **Stable** | **In remission** | **Unstable** |
| **Stable^1^** | 0.966 | 0.031 | 0.003 |
| **In remission^1^** | 0.331 | 0.661 | 0.008 |
| **Unstable^2^** | 0.175 | 0.803 | 0.022 |
| 1. **NSPT + AZ** | | | |
|  | **Stable** | **In remission** | **Unstable** |
| **Stable^1^** | 0.966 | 0.031 | 0.003 |
| **In remission^1^** | 0.331 | 0.661 | 0.008 |
| **Unstable^2^** | 0.249 | 0.729 | 0.022 |

Note: ^1^Transition probabilities obtained from Mdala et al.

^2^Transition probabilities derived from RR of the NMA

**Additional File 6 –** Parameters populated in the Markov model.

| **Parameters** | **Label** | **Description** |
| --- | --- | --- |
| Treatment strategies | 1 | Subgingival debridement alone |
|  | 2 | Subgingival debridement + (AMOX+MET) |
|  | 3 | Subgingival debridement + AZ |
|  | 4 | Subgingival debridement + AMPs |
| Transitional probabilities | tpA2A | Probability of patients remain in unstable periodontitis |
|  | tpA2B | Probability of patients transitioned from unstable to in remission periodontitis |
|  | tpA2C | Probability of patients transitioned from unstable to stable periodontitis |
|  | tpB2A | Probability of patients transitioned from in remission to unstable periodontitis |
|  | tpB2B | Probability of patients remain in in remission periodontitis |
|  | tpB2C | Probability of patients transitioned from in remission to stable periodontitis |
|  | tpC2A | Probability of patients transitioned from stable to unstable periodontitis |
|  | tpC2B | Probability of patients transitioned from stable to in remission periodontitis |
|  | tpC2C | Probability of patients remain as stable periodontitis |
| Direct medical costs (Public) | Pdmca0 | Cost of initial assessment for unstable patients |
|  | Pdmca1 | Cost of treating unstable patients during the first recall visit |
|  | Pdmca2 | Cost of treating unstable patients during the second recall visit |
|  | Pdmca3 | Cost of treating unstable patients during the third recall visit |
|  | Pdmca4 | Cost of treating unstable patients during the fourth recall visit |
|  | Pdmcb1 | Cost of treating in remission patients during the first recall visit |
|  | Pdmcb2 | Cost of treating in remission patients during the second recall visit |
|  | Pdmcb3 | Cost of treating in remission patients during the third recall visit |
|  | Pdmcb4 | Cost of treating in remission patients during the fourth recall visit |
|  | Pdmcc1 | Cost of treating stable patients during the first recall visit |
|  | Pdmcc2 | Cost of treating stable patients during the second recall visit |
|  | Pdmcc3 | Cost of treating stable patients during the third recall visit |
|  | Pdmcc4 | Cost of treating stable patients during the fourth recall visit |
| Direct medical costs (Private) | Prdmca0 | Cost of initial assessment for unstable patients |
|  | Prdmca1 | Cost of treating unstable patients during the first recall visit |
|  | Prdmca2 | Cost of treating unstable patients during the second recall visit |
|  | Prdmca3 | Cost of treating unstable patients during the third recall visit |
|  | Prdmca4 | Cost of treating unstable patients during the fourth recall visit |
|  | Prdmcb1 | Cost of treating in remission patients during the first recall visit |
|  | Prdmcb2 | Cost of treating in remission patients during the second recall visit |
|  | Prdmcb3 | Cost of treating in remission patients during the third recall visit |
|  | Prdmcb4 | Cost of treating in remission patients during the fourth recall visit |
|  | Prdmcc1 | Cost of treating stable patients during the first recall visit |
|  | Prdmcc2 | Cost of treating stable patients during the second recall visit |
|  | Prdmcc3 | Cost of treating stable patients during the third recall visit |
|  | Prdmcc4 | Cost of treating stable patients during the fourth recall visit |
| Drug costs (Public) | PAM | Cost of full dose AMOX+MET as adjunct to subgingival debridement |
|  | PAZ | Cost of full dose AZ as adjunct to subgingival debridement |
| Drug costs (Private) | PrAM | Cost of full dose AMOX+MET as adjunct to subgingival debridement |
|  | PrAZ | Cost of full dose AZ as adjunct to subgingival debridement |

**Additional File 7 -** Estimated average direct medical costs for treatments provided during the first year of periodontal treatment according to periodontal health states in public dental clinics.

| **No.** | **Treatments** | **Unit cost (in 2020 MYR)** | **Treatment proportion among patients with periodontitis** | | | **Source** |
| --- | --- | --- | --- | --- | --- | --- |
|  |  |  | **Stable** | **In remission** | **Unstable** |  |
| 1. | **Initial assessment and cause-related therapy** | | | | | |
|  | Specialist outpatient | 128.03 | 100% | 100% | 100% | a (Page 227) |
|  | Full mouth periodontal assessment | 99.66 | 100% | 100% | 100% | b |
|  | Electric pulp test | 76.66 | 10% | 10% | 50% | b |
|  | Orthopantomograph | 74.69 | 0% | 0% | 100% | a (Page 310) |
|  | Periapical radiograph | 896.24 | 50% | 50% | 100% | a (Page 310) |
|  | Oral hygiene instruction | 76.66 | 100% | 100% | 100% | b |
|  | Subgingival debridement (full mouth) | 679.65 | 0% | 0% | 50% | a (Page 250) |
|  | Subgingival debridement (per quadrant) | 1796.75 | 0% | 0% | 50% | a (Page 250) |
|  | **Treatment costs** |  | **760.15** | **760.15** | **2551.82** |  |
| 2. | **First recall visit** |  |  |  |  |  |
|  | Specialist outpatient | 128.03 | 100% | 100% | 100% | a (Page 227) |
|  | Periodontal reassessment | 132.52 | 100% | 100% | 100% | b |
|  | Oral hygiene reinforcement | 83.23 | 100% | 100% | 100% | b |
|  | Scaling and polishing as indicated | 456.66 | 50% | 100% | 100% | a (Page 246) |
|  | Subgingival debridement (full mouth) | 679.65 | 0% | 0% | 20% | a (Page 250) |
|  | Subgingival debridement (per quadrant) | 1796.75 | 0% | 50% | 80% | a (Page 250) |
|  | **Treatment costs** |  | **572.12** | **1698.82** | **2373.78** |  |
| 3. | **Second recall visit** |  |  |  |  |  |
|  | Specialist outpatient | 128.03 | 100% | 100% | 100% | a (Page 227) |
|  | Periodontal reassessment | 132.52 | 100% | 100% | 100% | b |
|  | Oral hygiene reinforcement | 83.23 | 100% | 100% | 100% | b |
|  | Scaling and polishing as indicated | 456.66 | 50% | 100% | 100% | a (Page 246) |
|  | Subgingival debridement (full mouth) | 679.65 | 0% | 0% | 20% | a (Page 250) |
|  | Subgingival debridement (per quadrant) | 1796.75 | 0% | 50% | 80% | a (Page 250) |
|  | **Treatment costs** |  | **572.12** | **1698.82** | **2373.78** |  |
| 4. | **Third recall visit** |  |  |  |  |  |
|  | Specialist outpatient | 128.03 | 100% | 100% | 100% | a (Page 227) |
|  | Periodontal reassessment | 132.52 | 100% | 100% | 100% | b |
|  | Oral hygiene reinforcement | 83.23 | 100% | 100% | 100% | b |
|  | Scaling and polishing as indicated | 456.66 | 50% | 100% | 100% | a (Page 246) |
|  | Subgingival debridement (full mouth) | 679.65 | 0% | 0% | 20% | a (Page 250) |
|  | Subgingival debridement (per quadrant) | 1796.75 | 0% | 50% | 80% | a (Page 250) |
|  | **Treatment costs** |  | **572.12** | **1698.82** | **2373.78** |  |
| 5. | **Fourth recall visit** |  |  |  |  |  |
|  | Specialist outpatient | 128.03 | 100% | 100% | 100% | a (Page 227) |
|  | Periodontal reassessment | 132.52 | 100% | 100% | 100% | b |
|  | Fluoride varnish application | 106.70 | 50% | 0% | 0% | a (Page 248) |
|  | Oral hygiene reinforcement | 83.23 | 100% | 100% | 100% | b |
|  | Scaling and polishing as indicated | 456.66 | 50% | 100% | 100% | a (Page 246) |
|  | Subgingival debridement (full mouth) | 679.65 | 0% | 0% | 20% | a (Page 250) |
|  | Subgingival debridement (per quadrant) | 1796.75 | 0% | 50% | 80% | a (Page 250) |
|  | Periodontal surgery | 1556.69 | 0% | 0% | 50% | a (Page 389) |
|  | **Treatment costs** |  | **625.46** | **1698.82** | **3152.12** |  |

1. Fees (Medical) (Cost of Services) Order 2014 (P.U.(A) 363/2014)
2. Local study by Mohd-Dom et al. (2016)

| **Medication costs:**  AMOX+MET: MYR 8.40 (Consumer Price Guide MOH)  AZ: MYR6.90 (Consumer Price Guide MOH) | **Adjustment to 2020 prices**  2012 GDP deflator 96.24960076  2014 GDP deflator 98.79660232  2020 GDP deflator 105.4115695  Source: https://databank.worldbank.org/source/world-development-indicators# |
| --- | --- |

**Additional File 8 -** Estimated average direct medical costs for treatments provided during the first year of periodontal treatment according to periodontal health states in private dental clinics.

| **No.** | **Treatments** | **Unit cost (in MYR 2020)** | **Treatment proportion among patients with periodontitis** | | | **Source** |
| --- | --- | --- | --- | --- | --- | --- |
|  |  |  | **Stable** | **In remission** | **Unstable** |  |
| 1. | **Initial assessment and cause-related therapy** |  |  |  |  |  |
|  | Consultation + Examination + Treatment plan | 200.00 | 100% | 100% | 100% | a & b |
|  | Orthopantomograph | 212.50 | 0% | 0% | 100% | a & b |
|  | Periapical radiograph | 900.00 | 50% | 50% | 100% | a & b |
|  | Subgingival debridement (Full mouth) | 2350.00 | 0% | 0% | 50% | b |
|  | Subgingival debridement (per quadrant) | 5000.00 | 0% | 0% | 50% | a |
|  | **Treatment costs** |  | **650.00** | **650.00** | **4987.50** |  |
| 2. | **First recall visit** |  |  |  |  |  |
|  | Consultation + Examination + Treatment plan | 200.00 | 100% | 100% | 100% | a & b |
|  | Subgingival debridement (Full mouth) | 2350.00 | 0% | 0% | 20% | b |
|  | Subgingival debridement (per quadrant) | 5000.00 | 0% | 50% | 80% | a |
|  | Scaling and polishing as indicated | 277.50 | 50% | 100% | 100% | a & b |
|  | **Treatment costs** |  | **338.75** | **2977.50** | **4947.50** |  |
| 3. | **Second recall visit** |  |  |  |  |  |
|  | Consultation + Examination + Treatment plan | 200.00 | 100% | 100% | 100% | a & b |
|  | Subgingival debridement (Full mouth) | 2350.00 | 0% | 0% | 20% | b |
|  | Subgingival debridement (per quadrant) | 5000.00 | 0% | 50% | 80% | a |
|  | Scaling and polishing as indicated | 277.50 | 50% | 100% | 100% | a & b |
|  | **Treatment costs** |  | **338.75** | **2977.50** | **4947.50** |  |
| 4. | **Third recall visit** |  |  |  |  |  |
|  | Consultation + Examination + Treatment plan | 200.00 | 100% | 100% | 100% | a & b |
|  | Subgingival debridement (Full mouth) | 2350.00 | 0% | 0% | 20% | b |
|  | Subgingival debridement (per quadrant) | 5000.00 | 0% | 50% | 80% | a |
|  | Scaling as indicated | 277.50 | 50% | 100% | 100% | a & b |
|  | **Treatment costs** |  | **338.75** | **2977.50** | **4947.50** |  |
| 5. | **Fourth recall visit** |  |  |  |  |  |
|  | Consultation + Examination + Treatment plan | 200.00 | 0% | 100% | 100% | a & b |
|  | Maintenance phase | 350.00 | 100% | 0% | 0% | a |
|  | Subgingival debridement (Full mouth) | 2350.00 | 0% | 0% | 20% | b |
|  | Subgingival debridement (per quadrant) | 5000.00 | 0% | 50% | 80% | a |
|  | Scaling and polishing as indicated | 277.50 | 50% | 100% | 100% | a & b |
|  | Periodontal surgery | 1875.00 | 0% | 0% | 50% | a & b |
|  | **Treatment costs** |  | **488.75** | **2977.50** | **5885.00** |  |

1. UMCS Fee schedule
2. Interview with private practitioners

| **Medication costs:**  AMOX+MET: MYR40.00/complete dosage  AZ: MYR40.00/complete dosage | **Adjustment to 2020 prices**  2012 GDP deflator 96.24960076  2014 GDP deflator 98.79660232  2020 GDP deflator 105.4115695  Source: https://databank.worldbank.org/source/world-development-indicators# |
| --- | --- |
